# Longitudinal data on cortical thickness before and after working memory training

**DOI:** 10.1016/j.dib.2016.03.090

**Published:** 2016-04-02

**Authors:** Claudia Metzler-Baddeley, Karen Caeyenberghs, Sonya Foley, Derek K. Jones

**Affiliations:** a Cardiff University Brain Research Imaging Centre (CUBRIC), School of Psychology, and Neuroscience and Mental Health Research Institute (NMHRI), Cardiff University, Cardiff CF10 3AT, UK; b School of Psychology, Australian Catholic University, Melbourne, Australia

**Keywords:** Cortical thickness, Subcortical volume, Working memory training, Supplementary information

## Abstract

The data and supplementary information provided in this article relate to our research article “Task complexity and location specific changes of cortical thickness in executive and salience networks after working memory training” (Metzler-Baddeley et al., 2016) [1]. We provide cortical thickness and subcortical volume data derived from parieto-frontal cortical regions and the basal ganglia with the FreeSurfer longitudinal analyses stream (http://surfer.nmr.mgh.harvard.edu [2]) before and after Cogmed working memory training (Cogmed and Cogmed Working Memory Training, 2012) [3]. This article also provides supplementary information to the research article, *i.e.*, within-group comparisons between baseline and outcome cortical thickness and subcortical volume measures, between-group tests of performance changes in cognitive benchmark tests (www.cambridgebrainsciences.com [4]), correlation analyses between performance changes in benchmark tests and training-related structural changes, correlation analyses between the time spent training and structural changes, a scatterplot of the relationship between cortical thickness measures derived from the occipital lobe as control region and the chronological order of the MRI sessions to assess potential scanner drift effects and a *post-hoc* vertex-wise whole brain analysis with FreeSurfer Qdec (https://surfer.nmr.mgh.harvard.edu/fswiki/Qdec [5]).

## Specifications Table

**Table:** 

Subject area	*Psychology*
More specific subject area	*Cognitive Neuroscience, Brain plasticity, Cognitive training*
Type of data	*Tables, figures, images of results of qdec analysis*
How data was acquired	*Magnetic resonance imaging and cognitive assessment. Cortical thickness and subcortical volumes derived with FreeSurfer.*
Data format	*Analyzed*
Experimental factors	*Longitudinal randomized controlled intervention study*
Experimental features	*Longitudinal randomized controlled intervention study comparing the effects of adaptive working memory training relative to non-adaptive control activities (n=20 healthy adults per group) on cognition and MRI derived cortical thickness indices in cognitive control networks.*
Data source location	*Cardiff University Brain Research Imaging Centre, Cardiff, UK*
Data accessibility	*Data is provided in this article*

## Value of the data

• Transparency and comparability of research results.
• Calculation of effect sizes for future *apriori* sample size and power calculations.
• Information about potential confounding factors that may affect the interpretation of similar studies.

## 1 Data

We provide data on cortical thickness and subcortical volume in regions of interest of cognitive control networks [1]. These structural data were acquired on a 3 Tesla GE Magnetic Resonance Imaging (MRI) system [1] and were derived with the FreeSurfer longitudinal analyses stream [2].

## 2 Experimental design, materials and methods

**Fig. 1:**
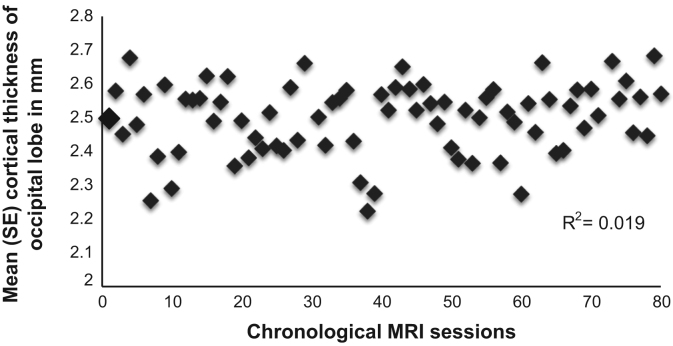
Plots the relationship between average cortical thickness measures in the right occipital lobe as a control region that was not expected to change with the intervention and the chronological order of the acquired MRI scans. There was no evidence of a drift in scanner acquisition across the MRI sessions.

48 healthy participants (19–40 years of age) were pseudo-randomly (with the provision to match groups for age and sex) allocated to an adaptive training or an active control group [1]. Both groups underwent structural MRI scanning on a 3 Tesla GE system at the Cardiff University Brain Research Imaging Center (CUBRIC) as well as cognitive assessment [4] before and after two months of working memory training (40 sessions in total) [3]. The training group performed the working memory tasks in an adaptive way, *i.e.*, training demands increased with performance levels whilst the control group performed the same tasks but in a non-adaptive way, *i.e.*, task difficulty was held at a low level and never exceeded an item span of 3. Participants could train from home and their progress and compliance was monitored throughout the training time. Eight participants dropped out so the final sample size for both groups was *n*=20 each (*n*=40 in total). Training-related changes in working memory span and executive functioning as well as in cortical thickness and subcortical volume in regions of interest of cognitive control networks (executive, salience, basal ganglia networks) were assessed. Cortical thickness and subcortical volume indices were derived with the FreeSurfer longitudinal analyses stream [2]. Training-specific effects were investigating with group by time interaction effects in the structural and cognitive outcome measures [6], [7] (Tables 1 and 2). Brain-function relationships and potential artefacts in the MRI data due to scanner drift effects were studied with correlational analyses (Fig. 1 and Tables 3 and 4).

### 2.1 Vertex-wise whole brain analysis of group and time effects on cortical thickness in FreeSurfer Qdec [5]

**Fig. 2:**
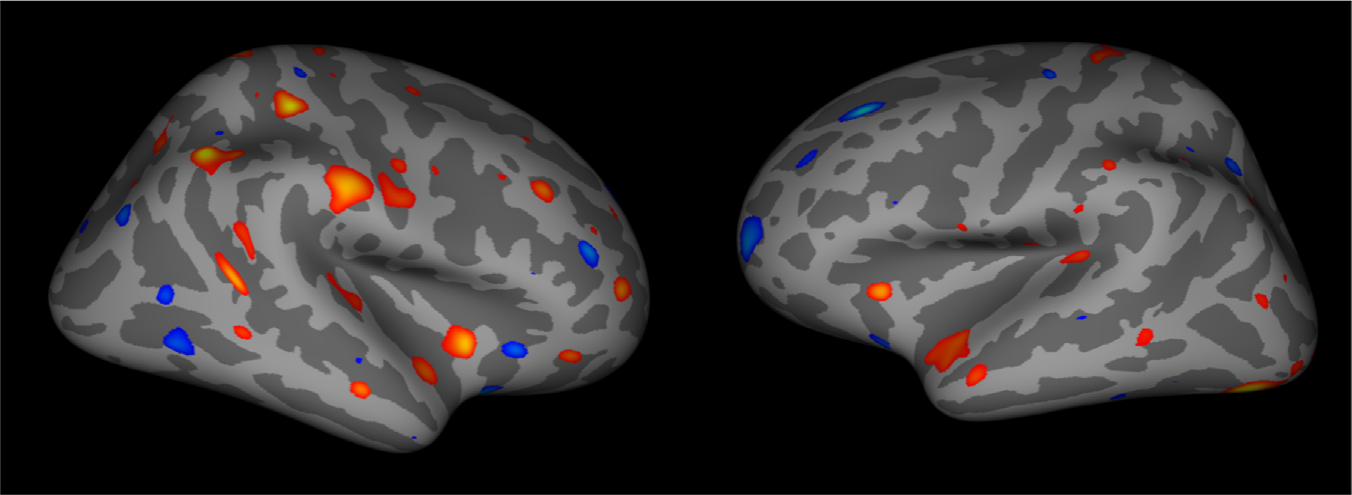
Displays lateral views on the right and left cortical surface respectively with clusters of regions for which a group effect across time was detected.

We conducted a *post-hoc* whole brain vertex-wise analyses in Qdec to test for the effects of group (adaptive training *versus* active control group) and time of assessment effects (baseline *versus* outcome) on cortical thickness measures. Cortical thickness indices were derived from the T_1_-weighted anatomical images smoothed with a kernel of 10. Multiple comparisons were controlled with a False Discovery Rate (FDR) of 5%. There were regions on both hemispheres with significant clusters of group effects across time (see Fig. 2) but no region demonstrated main effects of time or interaction effects between time and group, in either the right or the left hemisphere.

## Figures and Tables

**Table 1:** The results of the *post-hoc* paired *t*-tests [*t*(19)-value and *p*-value in brackets] comparing average cortical thickness indices before and after the two months training for each group (adaptive training and control group) separately.

	**Training (*n*=20)**	**Control (*n*=20)**
	***t*(19)-statistic (*p*-value)**	
Right *pars triangularis*	0.89 (0.38)	4.1 (0.001)
Right *pars opercularis*	1.44 (1.67)	1.5 (0.14)
Right caudal middle frontal	2.26 (0.03)	1.1 (0.29)
Left pallidum	2.53 (0.026)	0.36 (0.72)
Right insula	2.28 (0.03)	0.29 (0.77)
Left anterior cingulate	1.9 (0.07)	0.95 (0.35)

**Table 2:** Summary of non-significant results of independent *t*-tests of absolute changes (difference scores between post and pretraining performance scores) in performance in cognitive benchmark tests.

	***t*(38)-value**	***p*-value**
Double trouble	0.48	0.64
Grammatical reasoning	−1.13	0.26
Tree task	−0.65	0.52
Odd one out	0.71	0.48
Self-ordered search	0.15	0.88
Automated symmetry span	1.16	0.25

**Table 3:** Spearman׳s rho correlation coefficient *ρ* (*p*-values) between performance changes in the backwards digit span and spatial span tasks and changes in cortical thickness in the right caudal middle frontal gyrus, the right *pars triangularis* and the right insula and changes in subcortical volume in the left pallidum for the training and the control group (*n*=20).

	Backwards digit span	Spatial span
*Training group*		
Right caudal middle frontal	0.03 (0.9)	0.02 (0.92)
Right *pars triangularis*	−0.11 (0.64)	−0.01 (0.95)
Right insula	−0.63 (0.003)	0.27 (0.24)
Left pallidum	−0.32 (0.17)	0.37 (0.11)
*Control group*		
Right caudal middle frontal	0.32 (0.16)	−0.08 (0.74)
Right *pars triangularis*	−0.09 (0.69)	0.32 (0.16)
Right insula	−0.18 (0.43)	−0.11 (0.66)
Left pallidum	−0.11 (0.64)	−0.18(0.45)

**Table 4:** The Pearson correlation coefficients *r* (*p*-value) between the average time spent on training and changes in cortical thickness/subcortical volume across all regions of interest. There were no significant correlations at Bonferroni corrected level of significance (*p*<0.0015).

**Pearson correlation coefficient *r* (*p*-value)**	Average active time per training session
*Change in cortical thickness in ROIs on left hemisphere*	
Caudal anterior cingulate	0.041 (0.8)
Caudal middle frontal	−0.028 (0.86)
Inferiorparietal	0.234 (0.15)
Parsopercularis	0.202 (0.21)
Parsorbitalis	0.232 (0.15)
Parstriangularis	0.122 (0.45)
Rostral anterior cingulate	−0.047 (0.77)
Rostral middle frontal	0.210 (0.19)
Superior frontal	−0.036 (0.83)
Superior parietal	0.154 (0.34)
Supramarginal	0.115 (0.48)
Insula	0.092 (0.57)
*Change in cortical thickness ROIs on right hemisphere*	
Caudal anterior cingulate	−0.0289 (0.07)
Caudal middle frontal	0.248 (0.12)
Inferiorparietal	0.321 (0.04)
Parsopercularis	0.099 (0.54)
Parsorbitalis	0.205 (0.20)
Parstriangularis	0.208 (0.19)
Rostral anterior cingulate	0.042 (0.79)
Rostral middle frontal	0.109 (0.50)
Superior frontal	0.177 (0.27)
Superior parietal	0.198 (0.22)
Supramarginal	0.095 (0.55)
Insula	−0.194 (0.23)
*Change of subcortical volume in ROIs on left hemisphere*	
Thalamus	0.114 (0.49)
Caudate	0.108 (0.51)
Putamen	0.207 (0.19)
Pallidum	−0.194 (0.23)
*Changes of subcortical volume in ROIs on right hemisphere*	
Thalamus	0.398 (0.01)
Caudate	0.239 (0.14)
Putamen	0.201 (0.21)
Pallidum	−0.114 (0.48)
