# Supplementary material for: Longitudinal data on cortical thickness before and after working memory training
Source: Data Brief. 2016 Apr 2;7:1143–7. doi: 10.1016/j.dib.2016.03.090 (PMC4833123; doi:10.1016/j.dib.2016.03.090)
Supplement: Supplementary file 1 — Supplementary material [file mmc1.docx]

*Data article*

**Title: Supplementary data to research article “**Task complexity and location specific changes of cortical thickness in executive and salience networks after working memory training”.

**Authors:** Claudia Metzler-Baddeley^1^, Karen Caeyenberghs^2^, Sonya Foley^1^, Derek K Jones

**Affiliations:**  ^1^Cardiff University Brain Research Imaging Centre (CUBRIC), School of Psychology, and Neuroscience and Mental Health Research Institute (NMHRI), Cardiff University, Cardiff, CF10 3AT, UK; ^2^School of Psychology, Australian Catholic University, Melbourne, Australia

**Contact email:** Metzler-BaddeleyC@cardiff.ac.uk

The authors have no financial or non-financial conflict of interest to declare.

Conflict of interest – none.
